# Editorial: Multilingualism and neurodevelopmental disorders

**DOI:** 10.3389/fpsyg.2023.1267023

**Published:** 2023-08-15

**Authors:** Ai Leen Choo, Sara Smith, Amy Pratt, Sibylla Leon Guerrero

**Affiliations:** ^1^Department of Communication Sciences and Disorders, Georgia State University, Atlanta, GA, United States; ^2^Department of Language, Literacy, Ed.D., Exceptional Education, and Physical Education (LLEEP), University of South Florida, Tampa, FL, United States; ^3^Department of Communication Sciences and Disorders, University of Cincinnati, Cincinnati, OH, United States; ^4^Department of Education, University of California, Irvine, Irvine, CA, United States

**Keywords:** multilingualism, neurodevelopmental disorders, cognition, speech and language disorders, children, autism spectrum disorders (ASD), stuttering

Neurodevelopmental disorders encompass a range of diagnoses that commonly manifest as cognitive, speech, and language impairments (for a review, see Ismail and Shapiro, 2019). Symptoms of these disorders typically emerge in early development and become more conspicuous with age, as cognitive, speech and language demands increase (American Psychiatric Association, 2013). These disorders can have long-term and wide-ranging consequences, from difficulties with daily and social functioning to academic skills (Pratt, 2007; Kasari et al., 2011; Arnold et al., 2020).

There is growing evidence that multilingualism impacts cognitive development in the short- and long-term (Antoniou et al., 2016; Quinteros Baumgart and Billick, 2018; however see Paap and Greenberg, 2013). For example, typically developing multilingual children perform better on working memory tasks compared to monolingual children under certain circumstances (Morales et al., 2013). However, the development of children with neurodevelopmental disorders who are also multilingual is less well understood, particularly compared to monolinguals, due in part to a paucity of research and inconsistencies in how multilingualism is defined.

In this Research Topic, we present a set of articles focused on neurodevelopmental disorders in multilingual children. The first paper is a systematic review of bilingualism nomenclature in studies of children with autism spectrum disorder (ASD; Hantman et al.). The second paper examines the correlation between symptom severity, academic success, and socioemotional functioning using a national database (Foster et al.). Next, Gahl examines differences in stuttering identification among monolingual and multilingual children in the Early Childhood Longitudinal Study (ECLS) dataset. The last article investigates the effects of computerized cognitive training in children with an intellectual developmental disorder (Wu et al.). The topics provide important insights into the relationship between multilingualism and neurodevelopmental disorders and potential directions for translational research and treatment.

Hantman et al.'s systematic review of 103 articles found that multilingualism is not consistently labeled and defined across studies examining ASD. In addition to the commonly used *bilingual* or *multilingual*, a range of labels including *minority language, bilingual exposed*, and *heritage language* have been used to describe this population. Strikingly, a majority of studies failed to provide an operational definition of bilingualism. Even where provided, definitions of these labels varied widely (e.g., language learning experience in school settings, limited English proficiency). Further, nearly a third of the studies in their review did not report participants' age. Less than a fifth included their participants' history of language acquisition or language exposure. The authors suggest that these inconsistencies may lead to challenges in group categorization, translating findings, and consolidating analyses. This study highlights the gaps and issues in understanding the relationship between multilingualism and both ASD neurodevelopmental disorders more broadly.

Foster et al. switch our focus to the consequences of speech-language disorders in multilingual children. This study examined parent reports from the National Survey of Children's Health to understand how symptom severity influences academic success and socioemotional functioning. Parents of multilingual children were more likely to report more severe symptoms and higher rates of comorbidity relative to parents of monolingual children with speech-language disorders. The authors propose that speaking multiple languages may impact perceptions of language difficulty and make symptoms of some disorders more conspicuous, thus increasing parent reports. However, the study also found that multilingual children were not more likely to experience academic and socioemotional difficulties compared to their monolingual peers. Regardless of language status, more severe symptoms increased the probability of children experiencing academic difficulties and difficulty making friends. Further, rates of speech-language disorders were higher in males compared to females in both multilingual and monolingual children, consistent with past reports (e.g., McKinnon et al., 2007; Choo et al., 2022). Overall, this study highlights the differences and similarities between multilingual and monolingual children with speech-language disorders that could inform screening and classroom decisions.

Gahl explores the accuracy of stuttering identification in the ECLS, a longitudinal national survey of elementary school children. The author found inconsistent trends that do not align with conventional clinical findings for children with a home language other than English. Compared to monolingual-based criteria, the overall ratio of males to females who stutter was lower and did not increase with age as would be expected with higher recovery rates in females. Rather, the prevalence of stuttering across sex increased with age, conflicting with prior clinical findings. This study points to the challenges in accurately identifying neurodevelopmental disorders including stuttering, particularly in bilinguals where speech characteristics differ from monolingual-based criterion.

In the final study, Wu et al. investigated the efficacy of computerized training in 4 to 6.5 year old children with intellectual developmental disorders. Children received 5 weeks of training on visual perception, attention, memory and reasoning, either via an experimental computer program or with a trained therapist as a control condition. Both groups showed improved performance on intelligence and adaptive behavior assessments following the five-week training and largely maintained these improvements at 3-month follow-up. Interestingly, children trained through the computerized program outperformed those who received training from a therapist. The authors suggest that long-term intervention may be required to maintain the improvements, at least for some skills. The findings from this study are encouraging and make a case for computerized training in children with intellectual developmental disabilities.

Authors in this Research Topic share crucial work that highlights the gaps and challenges in understanding the relationship between multilingualism and neurodevelopmental disorders (Hantman et al.), the obstacles faced by children who are affected (Foster et al.), challenges in identifying children with neurodevelopmental disorders (Gahl), and the potential of treatments for this population (Wu et al.). The implications for future research are clear. The most fundamental challenge is the need for clear definitions and descriptions of multilingualism in research studies, without which valuable insights could be lost, and comparability across studies impeded. More research is warranted to determine the impact of neurodevelopmental disorders on multilingual children. Supporting the needs of multilingual children requires a multifaceted approach to treatment research that accounts for linguistic and cultural variability.

## Author contributions

AC: Conceptualization, Writing—original draft, Writing—review and editing. SS: Writing—review and editing. AP: Writing—review and editing. SL: Writing—review and editing.
